# Cardiac arrest during sports activity is difficult to recognise? Let the AED do the job!

**DOI:** 10.1007/s12471-018-1097-1

**Published:** 2018-03-08

**Authors:** A. Zorzi, D. Corrado

**Affiliations:** Department of Cardiac, Thoracic and Vascular Sciences, University of Padova, Padova, Italy

To the Editor,

We read with interest the article by Dr. Panhuyzen-Goedkoop et al. who reviewed videos of sudden cardiac arrest (SCA) of soccer players and analysed possible reasons why the condition was not promptly recognised and treated [1]. The footage of their fellow countryman who suffered SCA in 2017 while playing a friendly football match in Austria probably had an impact on our Dutch colleagues. The player of the Amsterdam team, Ajax, unfortunately sustained brain damage, possibly as a result of delayed resuscitation. In 2012, our medical community was also shocked by the SCA of two Italian professional athletes during competitive sports, including one Olympic medallist. In neither case was an automatic external defibrillator (AED) immediately used, despite the presence of medical personnel and an ambulance in both cases.

Reasons for delayed resuscitation may include the shock of an unexpected event and an attempt to open the airway. We have created a video (available at https://youtu.be/BmVUIAOTdGo) including selected footage from 24 cases of SCA or arrhythmic syncope that occurred in the last 15 years. These images suggest other possible causes. The first is that in many cases agonal breathing and involuntary limb movements are clearly visible after loss of consciousness. Such a scenario may confuse unexperienced first rescuers who may expect a SCA victim to be completely inanimate. The second is that an AED was either unavailable or used with delay in most cases. The video shows that on the few occasions when defibrillation was provided quickly, the athlete survived, confirming that this treatment markedly improves the outcome of out-of-hospital SCA [2]. Current resuscitation guidelines place high emphasis on using the AED as soon as it is available and less emphasis on airway management and rescue breathing. In particular, chest compression-only plus defibrillation are recognised as a valuable alternative to standard cardiopulmonary resuscitation for less experienced rescuers [3]. Despite updates in resuscitation guidelines, athletes continue to die during sports activities because of failure to implement the very first step in life support algorithms: recognition of SCA. However, if an AED is available within moments (as it should be in all sports facilities), this step may be unnecessary.

AEDs are highly reliable tools which analyse the heart rhythm and prompt rescuers to only deliver a shock if ventricular fibrillation or ventricular tachycardia (which are responsible for the vast majority of non-traumatic SCA) is diagnosed. Besides providing life-saving shocks, the majority of AEDs guide rescuers with voice and visual instructions through the different phases of resuscitation. As the automatic rhythm assessment takes only a few seconds, immediate AED use in any case of loss of consciousness, and not only after SCA is diagnosed, is unlikely to cause significant treatment delay. Hence, we believe that a clear indication should be given by first-aid instructors to medical personnel attending sport events: ‘*if an athlete falls to the ground and remains unresponsive, even if he/she appears to be breathing or moving, immediately bring the AED, switch it on, place the pads on the chest and just listen to the voice prompts*’.
